# Tumour treating fields in a combinational therapeutic approach

**DOI:** 10.18632/oncotarget.26344

**Published:** 2018-11-27

**Authors:** Joshua Branter, Surajit Basu, Stuart Smith

**Affiliations:** ^1^ Children’s Brain Tumour Research Centre, School of Medicine, University of Nottingham, Queen’s Medical Centre, Nottingham, UK; ^2^ Queen’s Medical Centre, Department of Neurosurgery, Nottingham, UK

**Keywords:** tumour treating fields, TTFields, combination therapy, optune, glioblastoma

## Abstract

The standard of care for patients with newly diagnosed Glioblastoma multiforme (GBM) has remained unchanged since 2005, with patients undergoing maximal surgical resection, followed by radiotherapy plus concomitant and maintenance Temozolomide. More recently, Tumour treating fields (TTFields) therapy has become FDA approved for adult recurrent and adult newly-diagnosed GBM following the EF-11 and EF-14 trials, respectively. TTFields is a non-invasive anticancer treatment which utilizes medium frequency alternating electric fields to target actively dividing cancerous cells. TTFields selectively targets cells within mitosis through interacting with key mitotic proteins to cause mitotic arrest and cell death. TTFields therapy presents itself as a candidate for the combinational therapy route due to the lack of overlapping toxicities associated with electric fields. Here we review current literature pertaining to TTFields in combination with alkylating agents, radiation, anti-angiogenics, mitotic inhibitors, immunotherapies, and also with novel agents. This review highlights the observed synergistic and additive effects of combining TTFields with various other therapies, as well highlighting the strategies relating to combinations with electric fields.

## INTRODUCTION

The use of electric fields for the treatment of neurological disorders pre-dates its use in the treatment of glioma [1]. Electric fields administered to the brain demonstrate profound effects specific to the parameters used – being frequency (Hertz – Hz), intensity (Volts – V) and pulse-width (Seconds – s). This review will focus on the Optune™ technology relevant to the treatment of brain tumours.

## OPTUNE™ (FORMERLY KNOWN AS NOVOTTF-100A)

The Optune system is a US Food and Drug Administration (FDA) approved novel anti-mitotic device that delivers continuous alternating electric fields to the patient for the treatment of primary and recurrent Glioblastoma multiforme (GBM). Optune is indicated for patients which are of at least 22 years of age, with histologically confirmed supratentorial GBM (WHO grade IV astrocytoma [2]). Optune in combination with Temozolomide (TMZ) has been approved for use in adult patients with newly diagnosed GBM following maximal safe resection, as well as completion of radiation therapy with concomitant TMZ [3].

**Figure 1:**
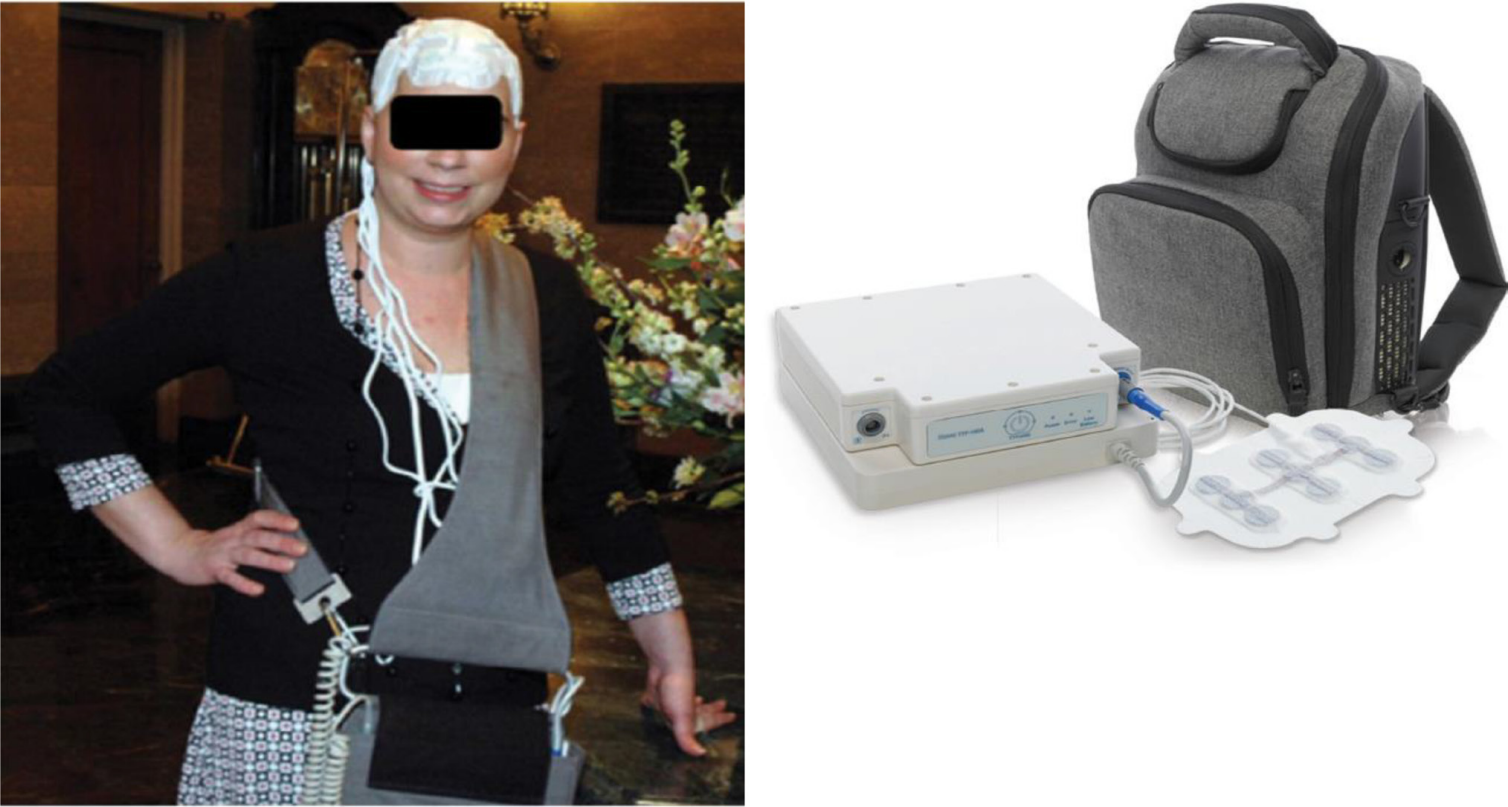
The Optune System (Left) The Optune System as worn by a patient. (Right) The Optune System consisting of a field generator connected to a transducer array, with the included backpack to facilitate portability of the field generator.

The Optune system is composed of four transducer arrays, a field-generator, and a power source (Figure 1). The field-generator delivers electric fields through the insulated transducer arrays which are applied to the shaved scalp of the patient. The field-generator delivers pre-set electric fields (200 kHz for glioma as determined by Kirson *et al.* [4] and with a minimum field intensity of 1.0 V/cm [5] – termed tumour treating fields (TTFields)) throughout the tumour in a non-invasive manner [6]. Much progress has also been made with optimisation of transducer layout in order to deliver a more efficacious treatment to improve patient outcome. The optimal array placement on the patient’s scalp is calculated using NovoTAL™ (Novocure Ltd., Haifa, Israel) simulation software, which will look to optimise field intensity within the tumour with variables such as tumour loci and patient’s head measurements [7].

A single transducer array is composed of 9 insulated biocompatible ceramic disks. A conductive hydrogel is applied to the patient’s shaven scalp to prevent direct contact of the ceramic disks and scalp. Thorough and frequent shaving of the patient’s scalp is required for optimal contact between the transducer arrays and skin. Application of the transducer arrays to the scalp of the patients is not a sterile process, however the prescribed transduced arrays are supplied in individual sterile packages in order to reduce risk of infection. For GBM patient’s, Optune TTFields therapy is delivered through two pairs of orthogonally positioned transducer arrays on the patient’s scalp. These particular components are secured in place, with emphasis on continuous skin contact, by being attached to a hypoallergenic medical adhesive bandage. A single cable connects each transducer array to the portable field generator component of the Optune system [6]. A critique of the Optune system was the cumbersome nature of the field-generator, however this has been addressed with the production of a second generation design – yielding a reduction in total weight of over 50% (https://www.optune.com/hcp/therapy/system).

A number of contraindications are associated with the Optune system which could discourage uptake. Firstly, the effects of TTFields have only been studied with adults, therefore Optune TTFields therapy may only be administered to patients of 22 years or older. Patients are excluded from treatment if they have a skull defect which would restrict attachment of the transducer arrays, and also if they have known sensitivity to conductive hydrogels (https://www.optune.com/Content/pdfs/Optune_IFU_8.5x11.pdf). Medically implanted devices (such as DBS devices) were removed from the list of official contraindications due to a retrospective analysis of 1,402 patients which revealed no device related safety concerns for the 49 patients with implanted medical devices [8]. Lastly, considerations have to be made regarding patients without access to assistance with the Optune system (either a friend/relative or carer) or do not have sufficient mental competence for personal maintenance of and compliance with the system, as patients are expected to comply to the system on average at least 18 hours per day [9].

## TTFIELDS MECHANISMS OF ACTION

Understanding the approach of TTFields requires familiarity with three concepts. Firstly, electric fields may be uniform – an electric field which is constant at every point in space, or non-uniform – an electric field which varies in magnitude and/or direction (convergent or divergent) at a given point in space [10]. Secondly, an electric field may be a constant field – where the source charge remains constant such that a test charge will converge, in a single direction, within the constant field towards the opposite polarity source. An electric field may also be a time-varying field – where the charges of the sources do not remain constant and therefore alternate space [10]. Lastly, the test charge may either be an electric monopole or an electric dipole. An electric monopole will simply alternate direction of travel along an alternating uniform field, however, an electric dipole will spin within the alternating uniform field while orientating with its current direction. Both electric monopoles and dipoles will travel towards the point of greatest electric field intensity within a converging non-uniform field – a process known as dielectrophoresis [10].

The concept of treating cancer through targeting dividing tumour cells with TTFields was originally raised by Prof. Yoram Palti, Israel Institute of Technology [4]. The initial theory was that mitotic activity of tumour cells could be disrupted with the application of alternating electrical fields and become a potential therapeutic avenue [4]. Palti and colleagues tested this hypothesis on eleven different cell lines, with multiple cancer types, and subsequently demonstrated that the formation of mitotic spindles could be disrupted by TTFields. More specifically, Palti and colleagues showed that TTFields interfered with polymerization of tubulin subunits, a necessary process within metaphase required for cell division [4]. Other notable conclusions of the study were the significance of optimising the TTFields, to a specific cancer type, to exhibit maximal effectiveness without the consequences of excessive tissue heating or stimulation. These parameters being an intermediate frequency (200 kHz) with a low intensity (1–3 V/cm) for glioma cell lines [4]. The considerations of these parameters is necessary to avoid unwanted membrane depolarization of excitable cells and tissue heating [11]. However, these undesirable stimulations greatly decline when the frequency of an alternating electric field increases above 1kHz because of the excitable membranes’ hyperpolarization/depolarization cycles becoming integrated due to a membrane’s excitability response time simply being too slow to handle such high frequencies [4, 11]. Conversely, exceptionally high frequencies, within the MHz range, cause greater integration of the hyperpolarization/depolarization cycles resulting in dielectric losses – electrical heating as a result of rapidly oscillating molecules [4, 11].

### Anti-mitotic effects of TTFields

As highlighted previously, living cells respond to electric fields due to intracellular polar molecules being susceptible to electrical manipulation. Of particular interest is the mechanism and interactions of TTFields with these polar molecules during mitosis, as this is where TTFields may express their anti-tumour effects [4, 12–14].

Firstly, the detailed intricacies of mitosis are beyond the scope of this review. However, certain events during this process are paramount to the understanding of how TTFields function and have therapeutic potential. Before the actualisation of the metaphase-plate, paired centromeres are captured by the ends of microtubules, before becoming orientated towards their respective poles by their opposing metaphase spindles, during anaphase to become separated via cytokinesis [12, 15]. Sister chromatid separation through cytokinesis is a consequence of Cyclin B and Securin ubiquitin-mediated degradation by Cdc20 and Anaphase Promoting Complex C (APC/C) [12, 15, 16]. This APC/CCdc20 destruction complex is dependent on proper microtubule localisation and function within the anaphase and metaphase spindles [12, 15, 16]. The key point of this process is that errors that disrupt this intricate process, particularly following commitment to anaphase, are likely to be irrevocable [17]. This dependency of cancer cells on mitotic competence is the basis for a number of therapies targetting mitosis [18], and is also the basis of TTFields as previously stated, errors committed within anaphase results in a multitude of cell fates and phenotypes including mitotic catastrophe, aberrant mitotic exit, aneuploidy, multi-nucleation, mitotic slippage and apoptosis [12, 14, 19].

One of the processes which TTFields target is tubulin polymerization [4, 10, 12, 14]. Microtubules consist of polymerized tubulin dimers, arranged around a hollow core in parallel [20]. The dynamic instability of microtubules is particularly crucial for cytoskeleton remodelling which occurs during mitosis, simply put, tubulin dimers undergo expeditious cycles of polymerisation and depolymerisation. Both α-tubulin and β-tubulin are bound to guanosine triphosphate (GTP) which in turn regulates the polymerization process, and it is the hydrolysis of GTP, bound to β-tubulin, to guanosine diphosphate which favours depolymerisation [20, 21]. In summary, microtubule formation is determined by the rate of tubulin polymerization relative to the rate of tubulin depolymerisation/GTP hydrolysis. Therefore, TTFields would promote depolymerisation, given that tubulins are among the most polar molecules within cells [10, 22], by causing misalignment of tubulin subunits as they become forced to align with the electric field rather than their respected microtubule filament axis [10, 22]. This in turn promotes hydrolysis of GTP to GDP at the positive end of the microtubule, dissociation of tubulin subunits and overall microtubule disruption [20].

Given that TTFields affect mitosis through their effects on proteins that possess a high dipole moment and significance within the mitotic process, it would be reasonable to assume that TTFields would interact with a large number of proteins. Recently, Gera *et al.* showed the effects of TTFields on other key mitotic proteins and that TTFields express a more diverse interaction than once thought [12]. Gera *et al.* focussed on the Septin heterotrimer (Referred to as Septin from now on), composed of Septin 2, 6 and 7, due to its large dipole moment of 2711D as well as its interaction with the cytokinetic cleavage furrow (CCF) formation [12]. Of note is the dipole moment of Septin which is larger than the dipole moment of tubulin dimers (1660D). Other components of the CCF were excluded from the analysis due to incomplete crystal structure information prohibiting dipole moment calculations, including Anillin and PLK1 [12]. The significance of Septin resides within the anaphase spindle midline and CCF, where through cooperation with Anillin, stabilisation of microtubule structures and the boundaries of the CCF contractility are distinguished [12, 23, 24]. Anillin functions as an adaptor protein for binding of ECT2 to the Septin/Anillin complex to facilitate both regulation and localisation of the CCF and for stability of anaphase spindle midlines [12, 24]. Septin/Anillin regulation of CCF contraction is through crosslinking actin, myosin II and RhoA [25, 26] to facilitate actin-dependent myosin contraction at the CCF [27]. In a similar fashion as tubulin, TTFields would exert rotational stress on Septin, and most likely many other proteins involved within CCF formation/regulation and progression through anaphase.

Lastly, in combination with previously discussed protein interactions, TTFields also disrupt mitosis through membrane blebbing at times coinciding with the onset of anaphase [10, 12, 22]. The most probable causes of such violent blebbing observed as a response to TTFields would be; i) aberrant localisation of CCF contractile elements, producing ectopic cleavage furrows [12] and ii) dielectrophoretic forces acted upon the CCF [4, 10, 12]. TTFields in combination with the mitotic cell’s morphological changes (a resemblance to an hourglass) during the formation of the two daughter cell produces a non-uniform intracellular electric field, with the highest electric field density at the CCF directly between the dividing cells [22]. As described previously, sufficiently polar organelles and other macromolecules will gravitate towards the point of the greatest electric field intensity – the CCF, further disrupting the intricate mitotic process. It has been noted that these mechanisms are responsible for membrane disruption analogous to membrane blebbing [4, 10, 12, 28].

Overall, these results demonstrate how TTFields appear to affect mitotic cells throughout the latter stages of/and subsequent to metaphase [12, 29]. Literature has also demonstrated that the anti-mitotic effects of TTFields operates in both a p53-dependent [12] and –independent [30] manner. Also, this presents TTFields as a ‘new wave’ mitotic inhibitor due to the metaphase/anaphase specific disruption paradigm, where other mitotic inhibitors and traditional therapies mediate anti-tumour effects by triggering the G1/S or G2/M checkpoints of the cell cycle [12, 31, 32]. This could potentiate a synergistic effect of TTFields with other therapies affecting mitosis in combination from a more ‘complete’ coverage of the mitotic cycle.

## TTFIELDS IN BRAIN TUMOUR CLINICAL TRIALS

The first-in-human pilot trial was conducted between 2004 and 2007 following encouraging *in vitro* and animal study data. The study assessed the safety and efficacy of TTFields therapy using the NovoTTF-100a system in 10 patients with recurrent GBM [13]. Overall; the patients had a median overall survival (OS) of 14.4 months, a 1-year survival rate of 67.5% and a median time until tumour progression of 6.0 months [13]. Notably, there were 2 patients who demonstrated an 84 and 87 month survival from TTFields therapy initiation with no radiological or clinical evidence of recurrent disease [33]. The most common side effect associated with the NovoTTF-100a system was contact dermatitis, which will be a recurrent theme with TTFields therapy, which results from hydrogel-induced localised irritation of the scalp underneath the transducer arrays of the NovoTTF-100a [33].

### Recurrent GBM (EF-11 Trial)

Following the trial conducted by Kirson *et al.*, [13], the pivotal phase III trial was conducted between 2006 and 2009 with the primary end-point being OS [34]. The trial was seeking to assess NovoTTF-100a as a monotherapy and to compare the treatment to best physician’s choice chemotherapy (BPC) for recurrent GBM patients (*n* = 237). The patients of the trial were randomized (1:1) to TTFields monotherapy (*n* = 120) or BPC (*n* = 117); patients of the BPC arm were administered either a single agent or combinational therapy regime containing; Bevacizumab (31%), Irinotecan (31%), BCNU/CCNU (25%), Carboplatin (13%), TMZ (11%), PCV (9%), other agents (7%) or none received (3%). Balance was achieved between treatment arms in regard to patient characteristics; 90% of patients were at second or more recurrence, the patient’s median age was 54 years and 19% of patients had previously been treated with Bevacizumab [34].

NovoTTF-100a therapy demonstrated very similar efficacy to the chemotherapies selected by the physicians, with the NovoTTF-100a arm having a median OS of 6.6 months compared to 6.0 months for the BPC arm (Hazard ratio (HR) = 0.86 [95% Confidence interval (CI), 0.66 – 1.12]; *p* = 0.27). Again, this was true for the progression-free survival (PFS) and the overall response rate. The PFS at 6 months was 21.4% for the NovoTTF-100a arm versus 15.1% for the BPC arm (HR = 0.81 [95% CI, 0.60–1.09]; *p* = 0.13), with the overall response rate being 14.0% and 9.6% (*p* = 0.19) for the NovoTTF-100a and BPC arms respectively [34]. However, differences between the treatment modalities become apparent when considering their respective safety profiles. A greater frequency of systemic toxicities, including Grade 3/4 haematological (17% of patients), gastrointestinal (17%), and infections (8%) was apparent in the BPC arm compared to the NovoTTF-100a demonstrating a 4%, 3% and 4% frequency of occurrence (*p* < 0.05; Fisher exact test) [34]. Quality of life was also observed to be higher in the NovoTTF-100a arm with regards to social, cognitive and emotional functioning, however, self-reported physical functioning was slightly worse than the BPC arm [34]. The decline of the NovoTTF-100a treated patients’ physical functioning may be due to the relatively cumbersome nature of the NovoTTF-100a system, as well as the high compliance requirements for effective TTFields therapy.

Completion of this Phase III trial and the subsequent post-hoc analyses [35, 36] gave some insight into therapeutic potential of TTFields. Firstly, NovoTTF-100a therapy has similar efficacy as chemotherapy for patients with recurrent GBM but with a far more favourable side-effect profile [34]. Secondly, compliance with the NovoTTF-100a system was the main predictor of improved OS for patients. Kanner *et al.* reported a significantly longer median OS for NovoTTF-100a treated patients when compliance of 75% or greater is achieved (i.e. mean compliance of 18 hours or more per day) with a median OS of 7.7 months for the ≥75% compliance patients versus 4.5 months for the <75% compliance patients (*p* = 0.042). Interestingly, this post hoc analysis also described a significant stepwise correlation between median OS and compliance, with median OS of 5.8, 6.0 and 7.7 months for <60%, 60%–79%, and 80%–99% compliance, respectively *(p =* 0.039) [35]. Lastly, this particular Phase III trial was the first and only trial to date directly comparing bevacizumab efficacy to another monotherapy in recurrent GBM patients.

### Newly-diagnosed GBM (EF-14 Trial)

Optune therapy was more recently tested in a Phase III trial for newly-diagnosed GBM patients after receiving their initial treatment as per the Stupp protocol [3]. The GBM patients (*n* = 700) were randomized 2:1 to either the TTFields with adjuvant TMZ or TMZ monotherapy arms, respectively. The primary end-point of the trial was achieved at the interim as PFS in the intent-to-treat (ITT) population was significantly greater for the TTFields plus TMZ arm versus the control. The secondary end-point of the trial would have been achieved if the median OS of the per-protocol treated population was significantly greater in the TTFields plus TMZ arm relative to the TMZ monotherapy arm [37]. Analysis of the first patients (*n* = 315) at the pre-specified interim after an 18 month minimum follow up demonstrated increases in median OS and PFS. Of note, the Independent Monitoring Committee for the trial recommended that the trial be terminated at the pre-specified interim due to perceived survival benefit of TTFields with TMZ. This resulted in the patients of the TMZ monotherapy arm being provided with access to Optune therapy [37].

The median OS for the TTFields plus TMZ arm was 19.6 months versus 16.6 months for the TMZ monotherapy arm (HR 0.75; log-rank *p* = 0.034) in the ITT population, and 20.5 months versus 15.5 months (HR 0.67; log-rank *p* = 0.0072) in the as per-protocol population. The median PFS in the ITT population was 7.1 months versus 4.0 months (HR 0.6; log-rank *p* = 0.0014) for the TTFields plus TMZ and the TMZ monotherapy arms, respectively [37]. As with the EF-11 trial, the addition of TTFields therapy did not produce any significant increases in systemic toxicities relative to chemotherapy alone. This was too be expected as TTFields were localised to the head, but similarly, this was associated with a significant increase in localized skin toxicities; 43% of patients receiving TTFields therapy experienced mild to moderate skin irritation, as well as 2% experiencing severe skin reactions (Grade 3) [37]. The overwhelming majority of these toxicities are skin rash/irritation based, and the nature and incidence rates of these toxicities in further studies are described here [38]. These TTFields associated dermatological toxicities may be managed prophylactically, as well as being treated should toxicities develop. The prophylactic approaches include, but are not limited to; frequent shifting of transducer array locations to minimize direct pressure to the scalp, particular care with application of transducer arrays on surgical scars, and maximising transducer array-skin contact may also reduce skin irritation. Once any dermatological toxicity develops, topical antibiotics and corticosteroids may be applied for infections or contact dermatitis and irritation, respectively [38]. A number of these dermatological complications arise from the repetitive application and removal of the transducer arrays, compounded by additional inflammation from the hydrogel used to cover the ceramic disc portion of the transducer arrays, as well as additional moisture from sweat [6, 38].

However, more recent reports on the trial’s mature data have shown improved patient survival following treatment, which follows trends seen at the interim analysis [37]. The median OS from initial randomisation is 20.9 months for TTFields/TMZ versus 16.0 months for TMZ alone treated patients (HR 0.63; log-rank *p* = < 0.01) [1]. Interestingly, this improvement to survival was also seen across 2, 3 and 4 year survival of patients in TTFields/TMZ versus TMZ alone; with the respective rates of 43% and 31%, 26% and 16%, and 13% and 5% (*p* = < 0.05 for all time points) [1].

## TTFIELDS IN COMBINATIONAL THERAPY

### Alkylating agents

In accordance with the current standard of care, TMZ is used in combination with RT for patient benefit [3]. Two relevant highlights of this randomized phase III are apparent; firstly, the combinational therapeutic route produced a significant increase to both median OS (12.1 to 14.6 months) and 2-year survival (10% to 27%), secondly, a genetic determinant of benefit from TMZ was present in the form of methylation status of O-6-methyl-guanine-DNA methyltransferase (MGMT) [3]. Similarly, phase III studies concerning elderly patients, showed that patients expressing a methylated MGMT status benefitted greatly from TMZ treatment, although not in combination with RT, further emphasises genetic predisposition to therapeutic response [39, 40]. MGMT functions to remove the highly mutagenic and genotoxic O^6^-methylgunanine residues caused by TMZ [41]. A negative feedback loop occurs upon MGMT-dependent repair of these methyl adducts whereby MGMT becomes irreversibly inactivated. It is in this absence of active MGMT that mismatch occurs during replication between methylgunanine and thymine and causes subsequent double-stranded breaks (DSBs) [41, 42]. Complimentary resistance mechanisms to TMZ, as well as other alkylating agents, are reviewed here [41].

Given the success of the initial trial, the EF-14 trial sought to evaluate the potential benefit of combining TTFields with TMZ compared to TMZ alone following chemoradiation in newly diagnosed GBM patients. As discussed previously, the current EF-14 trial data has shown promising additive efficacy of TTFields with TMZ [1, 37]. Silginer and colleagues investigated TTFields in combination with TMZ in pre-clinical glioma models focussing on MGMT-status and TMZ-resistance [43]. The study highlighted that TTFields’ efficacy was not dependent on MGMT expression, nor was it diminished within TMZ-resistant cell lines [43]. The lack of overlap between the TTFields and TMZ-resistance mechanisms is not wholly unexpected however, as the primary mechanism of action for TTFields has yet to be shown to interact with MGMT [14, 44]. This feature of the study highlights how TTFields may be an attractive therapeutic option for patients whom would not benefit greatly from TMZ treatment i.e. patients with a negative MGMT methylation status [44].

A proposed mechanism of synergism would be that TTFields may potentially influence DNA fragment orientation to perturb DSB-repair mechanisms [45]. A common theme will emerge that TTFields in combinational therapeutic routes appear to have positive synergistic effects without any known overlapping toxicities.

### Radiation therapy

Radiation therapy (RT) represents another physical treatment modality for cancer treatment. The high-energy ionizing radiation used for treatment damages the DNA of targeted cancerous cells, as well as normal cells which are adjacent to the targeted cells [46]. RT may be delivered to the patient through two different means; external beam radiation or through internal radiation sources, with external radiation being delivered via high-energy protons, photons or particle radiation [46]. Adjuvant RT would be utilised to target any residual tumour remaining following the resection albeit following a 4 week post-surgery recovery period [47]. As with other therapies, RT achieves therapeutic efficacy predominantly through a variety of DNA lesions; notably single- (SSBs) and DSBs [48]. It is these lesions which induce cell death via apoptosis and mitotic catastrophe [49, 50].

Given that TTFields influence polar molecules, TTFields should theoretically interact with the fragmented DNA strands following RT. Similar to how TTFields disrupt microtubule assembly [4, 10, 12, 14], TTFields may be able to influence DNA fragment orientation in a fashion to decrease DNA ligation to reduce the effectiveness of DNA repair mechanisms. This phenomenon has been reported on previously [45, 51] with reports of reduced clonogenic survival and cell viability despite the increased number of Rad51 and γ-H2AX foci when TTFields and radiation treatment were used in combination. This report suggests a reduction in DNA strand break repair competency due to the reduction in cell survival despite the upregulation of DNA repair markers Rad51 [52] and γ-H2AX [53]. More recently, the effects of TTFields in combination with radiation treatment were more intently interrogated, with an emphasis on timing TTFields with regards to RT [54, 55]. Kim and colleagues reported on the synergistic properties of TTFields with ionising radiation when TTFields were given prior to radiation treatment [54]. The combinational therapeutic route resulted in greater amounts of p53-dependent apoptosis, as well as produced mitotic anomalies indicative of TTFields treatment – multi-nucleated phenotype and both mono-polar and multi-polar spindle structures [14, 54]. Giladi and colleagues also investigated this combination but with TTFields following RT [55]. Again, it was shown that efficacy of RT may be increased with TTFields when administered following radiation treatment. Taken together, these reports suggest that TTFields may be used as a strategy to sensitise glioma to RT, whether TTFields is administered before or after RT. These interactions pose an interesting synergy paradigm which may be irrespective of established RT resistance and other DNA damage repair mechanisms.

### Anti-angiogenics

Bevacizumab is a humanised monoclonal antibody which antagonizes Vascular endothelial growth factor (VEGF) to inhibit binding to VEGF-Receptor (VEGFR) [56]. Bevacizumab acquired US Food and Drug Administration (FDA) approval for the treatment of recurrent GBM, following two Phase II studies – [57] and [58] in 2009. In summary, both studies concluded that Bevacizumab use was associated with a higher PFS. However, subsequent studies have shown that Bevacizumab does not significantly increase median OS when administered as a front-line therapy for newly diagnosed GBM patients [59, 60]. Furthermore, Bevacizumab has been associated with a number of negative side effects; decline in neurocognitive function, gastrointestinal perforation, thromboembolic events, renal failure, hypertension, neutropenia and overall decreased quality of life [56, 59–61]. It appears that the majority of the lower-grade adverse effects appear to be indicative of VEGF disruption in non-cancerous cells.

As with other combinational therapeutic routes, TTFields combined with Bevacizumab is intriguing as both have shown promise for patients, albeit Bevacizumab only for recurrent GBMs [56], and may provide additive efficacy. Firstly, TTFields efficacy has been shown in a large phase III trial to be comparable to BPC chemotherapies, including Bevacizumab (31% of patients), but without a diverse and adverse effects profile akin to conventional chemotherapies [34]. Lastly, there has been evidence to suggest that TTFields may increase the safety profile of Bevacizumab when used in combination for treatment of recurrent gliomas [62]. Although on a small scale, Elzinga *et al.* retrospectively analysed patients (n = 20) treated with the combination of TTFields with Bevacizumab and found no instances of intracranial haemorrhage or thromboembolic events [62]. These particular events occur in 3% and 2.4–12.5% of patients treated with Bevacizumab respectively [56], although it is worth noting that spontaneous intracranial haemorrhage occurs in roughly 2% of patients without Bevacizumab treatment [56]. However, the exact molecular nature, if any, of these synergistic events has yet to be elucidated and should be a topic of future research. Lastly, bevacizumab is known to reduce vasogenic brain oedema, thereby reducing patients’ dexamethasone requirements. The significance of reducing a patient’s dexamethasone’s dosage will be expanded upon in later sections.

### Mitotic inhibitors

Given the intricate, yet inherently unstable mitotic process in proliferative cells, many inhibitors have been explored for the treatment of cancers; including anti-microtubular, anti-kinase and other molecular targets. The induction of mitotic cell death (MCD) is the rationale behind targetting mitosis in cancer cells. Dividing cells are highly susceptible to MCD when exposed to disruptive stresses [63]. It is these stresses which have the potential to activate the spindle assembly checkpoint (SAC), leading to a prolonged mitotic arrest where a number of different cell fates are actualized, albeit intra- and inter-line variations in cell fates are expected [64]. Combining TTFields with SAC inhibitors has been investigated [65], and demonstrated increased levels of apoptosis and G2/M-phase accumulation of cells.

The anti-microtubular class of mitotic inhibitors can be divided into two distinct sub classes; microtubular-stabilizing (i.e. Taxanes and Epothilones) and microtubular-destabilizing agents (i.e. Vinca alkaloids) [66]. These agents have demonstrated anti-tumour activity in a variety of tumours such as breast, non-small cell lung and ovarian cancer [67]. The microtubule-stabilizers, such as Paclitaxel, typically bind β-tubulin with high affinity to induce conformational changes which in turn results in stability of tubulin interactions [66, 68]. Conversely, the microtubule-destabilizers, such as Vinblastine, target microtubule polymerization through binding the vinca domain of both tubulin monomers and microtubules, causing the necessary conformational changes to reduce microtubule formation [66, 68]. Although dissimilar, both subclasses aim to cause MCD in a SAC-dependent manner. However, there has been limited transfer from laboratory to clinical practice for the majority of mitotic inhibitors [67]. This may in part be due to two main limitations; i) the phenomenon of mitotic slippage to circumvent mitotic arrest/MCD and ii) G2/M selective inhibition. Firstly, the polyploid phenotype typical of cells following mitotic arrest presents a paradigm where multiple cell fates are a result, with cells either succumbing to MCD in the subsequent G1 phase, senescence or existing as viable multiploidal cells [69]. The latter has the potential to produce cells with increasing degrees of instability from subsequent cell cycles, an overall increase in cellular stress, chemoresistance and is a predictor of intrinsic taxane resistance [70, 71]. The selective nature of mitotic inhibitors naturally leads to limitations in drug efficacy. Considering drug retention times as well as mitotic-specific drugs targetting mitotic machinery at the G2/M-M-phase, a large population of G1- and S-phase cells may remain refractory to the cytotoxic treatment [67]. Compounding this selectivity, the mitotic index of human tumours has been estimated to be less than 1% with mean doubling times of a range of solid tumours ranging from ∼100 to ∼400 days summarized by Komlodi-Pasztor *et al.* [72]. These observations of substantial doubling times and low mitotic indexes of solid tumour emphasise the necessity of chronic treatment over a period of multiple months of mitotic inhibitors. However, significant dose-limiting toxicity has been associated with tubulin-targetting agents—notably neutropenia [67, 73]—which has been a persistent challenge during the drug development process. Therefore, improving drug half-life and/or drug delivery limitations, while simultaneously reducing the dose-limiting toxicities associated with tubulin-targetting agents, may be a promising area of investigation for improving mitotic inhibitory therapy for patients.

Mitotic kinase or associated protein inhibitors may also be a viable option for the treatment of cancer. More selective protein or kinase inhibitors seem to be attractive therapeutic options as they add more options for drug resistant tumours but have also been found to have less associated toxicities than their tubulin-associated counterparts on the whole [67, 72, 73]. Members of the Polo-like kinase (PLK) and Aurora kinase families are of particular interest to anti-mitotic therapies given their relatively restricted expression to M-phase, with minimal to null expression in G0, G1 and S-phases [73]. PLK1 and Aurora kinases are involved with multiple mitotic process including spindle assembly, cytokinesis, chromosome segregation and activation of the SAC [67, 74, 75]. Similar to microtubular associated therapies, inhibition of PLK1 and Aurora Kinase A in GBM cells appears to activate the SAC, cause MCD and mitotic arrest [76, 77]. However, given that both PLK1 and Aurora Kinase A expression appears at S-phase and peaks at the G2/M checkpoint [78, 79], combining these kinase inhibitors with the microtubule associated agents may provide greater therapeutic efficacy through a more complete coverage of the cell cycle.

TTFields may be considered as a physical novel mitotic inhibitor, so combining TTFields with biological mitotic inhibitors would appear logical. Firstly, assuming compliance to the Optune TTFields system is in the upper bracket of patient beneficial compliance (18 hours and above) [35], TTFields may overcome the limitations of mitotic inhibitor drug retention. Synergism between TTFields and mitotic inhibitors, particularly microtubule-stabilizers, has been demonstrated and novel mechanisms of increased efficacy have been postulated [80, 81]. Kirson *et al.*, suggested that as paclitaxel promotes microtubule elongation due to greater stability of tubulin dimers, TTFields would demonstrate greater influence over the microtubules due to the now greater dipole moment to increase microtubule misalignment [81, 82]. This is because the displacement vector of the positive to the negative charge is a function of an electric dipole moment. However, TTFields were shown to disrupt microtubules, through yet to be determined mechanisms, which may decrease paclitaxel efficacy in GBM [14]. More recently, Voloshin and colleagues investigated the potential for synergism of paclitaxel and TTFields in ovarian cancer cell lines [83]. The highlights of this study were the increased efficacy of TTFields when combined with paclitaxel, as well as increased accumulation of cells in the G2/M phase of the cell cycle when analysed with flow cytometry. However, accumulation of Caov-3 and OVCAR-3 cells in the G2/M phase of the cell cycle significantly increased relative to controls following 72 hours of TTFields treatment with A2780 cells fate being accumulation in the G1 phase following extended exposure to TTFields [83]. The differences between cell fates is further highlighted within the combination indexes (CI) of the cells; specifically, the A2780, OVCAR-3 and Caov-3 cell lines had Cis of 1.03, 0.81 and 0.86 respectively [83]. These data indicate a synergistic paradigm for the OVCAR-3 and Caov-3 cell lines but an additive effect for the A2780 cell line, however these observations may be due to differences in the intrinsic sensitivities of the cell lines to mitotic inhibitor treatment. Ovarian cells treated with TTFields in combination with paclitaxel also demonstrated multipolar spindle formations, which coincides with previous observations of TTFields treated cells [14].

It is conceivable the TTFields combined with microtubule-destabilizing agents, such as the vinca alkaloids, should produce a similar effect given their similar modes of action. This hypothesis gains credence from evidence of combinational therapy consisting of paclitaxel and vinorelbine, a semi-synthetic vinca alkaloid, significantly improving outcome for breast cancer patients [84]. Interestingly, evidence of combining the microtubule associated paradigms has also appeared in nature with both classes being present within the roots and rhizomes of the bat flower, *Tacca sp* [85]. Lastly, it has been shown that TTFields do not perturb localisation of PLK1 from its functional location at the anaphase spindle midline [12], but data regarding other mitotic associated proteins has yet to be collected. Therefore, there is reasoning behind combining TTFields with a number of mitotic inhibitors regardless of the mitosis stage of their action.

### Immunotherapy

Just as the previously detailed therapeutic options available to GBM patients, immunological agents may have the potential for synergism with TTFields, and thus improved efficacy. Although a newly emerging phenomenon, TTFields indeed seem to induce an immune response and its anti-tumour effects may be, at least in part, dependent on the competence of the patient’s immune system [29, 81, 86, 87]. Firstly, TTF-induced mitotic exit subjects the affected cells to cellular stress which among others, upregulates cell surface expression of calreticulin – an endoplasmic reticulum chaperone protein [88], a downregulation of anti-phagocytic signalling molecules such as the cell surface CD47 [89], as well as promotes secretion of HMGB1 in order to produce an immunogenic phenotype [90]. This response termed ‘Immunogenic cell death’ is a documented phenomenon of cancer cells when subjected to TTFields, which is dissimilar to the inherently immunosuppressive apoptosis [88, 90]. There is evidence in favour of TTFields promoting anti-tumour immunogenicity *in vitro* and *in vivo* [81, 86, 87]. Kirson *et al.* demonstrated how TTFields may inhibit metastasis to the lungs of solid tumours but also noted that significantly greater amounts of infiltrative immune cells were found intratumourally in the metastasis [87]. Immune cells bearing the markers CD4, CD8 and CD45 were among the infiltrative cells, inferring a T-cell mediated response, but this was only true for the TTFields treated rabbits as opposed to the TTFields treated mice [87]. A few potential reasons for this discrepancy are apparent: i) species differences; ii) cancer cell line differences; iii) tumour volume differences; iv) TTFields treatment duration differences. Naturally, differences in cell lines used, as well as species would equate to differences in efficacy of treatments and competency of the immune system [91]. Lastly, significant differences in exposure durations to TTFields (1 week for mice and 5 weeks for rabbits) would account for differences in response, and indeed highlights a potential dose-dependent relationship between TTFields and an effective immune response. The significance of this dose-dependent relationship was highlighted in both the EF-11 [34] and EF-14 [1] human trials, so it should be expected that longer treatment duration should result in improved treatment outcome.

Wong *et al.* 2014 had previously observed that patients with previous low-grade glioma histology and low dosing of dexamethasone in the Phase III trial examining response rates of NovoTTF-100A as a monotherapy relative to the best physicians choice (BPC) chemotherapy had a more favourable outcome [92]. Although, it is well recognised that patients with a secondary-GBM, have a significantly more favourable prognosis and longer survival [93]. Neuro-oncologists traditionally use dexamethasone for patients with malignant brain tumours for its anti-oedema effects [94]. However, dexamethasone does exhibit profound immunosuppressive influence over patients [94], and therefore has the potential to reduce efficacy of TTFields. Wong *et al.* 2015 further examined the effect of dexamethasone on patients and determined a threshold of dexamethasone exposure for preferential survival with TTFields treatment [86]. Using an unsupervised mathematical algorithm, it was determined that patients receiving over 4.1mg/day had a 2.3-fold decrease in median OS for the TTFields treated cohort, compared to a 1.5 fold decrease in median OS for the BPC chemotherapy treated cohort [86]. A decrease in median OS was also seen with a progressive decrement in both cohorts until about 8.0 mg/day was achieved where there was no further significant effect on median OS [86]. However, it could also be assumed that patients requiring higher doses of dexamethasone may be stratified as higher-risk patients, so may therefore have a lower expected OS irrespective of TTFields.

Given that TTFields have not been shown to have any consequential effects on immune system competence, unlike traditionally used therapeutics [95], TTFields combined with immunotherapeutics gain credence for a number of reasons. Firstly, as stated above TTFields do not compromise the immune system as other agents do, potentially reducing the required dose of concurrent therapeutics. This dosage reduction should in turn reduce their inherent immune compromising nature. Secondly, the physical nature of TTFields appears to improve the infiltrative capacity of CD4 and CD8 cells in rabbit models [87], this should have clear synergistic effects with immunotherapeutics such as dendritic cells [96]. However, this potential synergism has yet to be studied and may be the key to bring the promising field of immunotherapeutics closer to the clinic for GBM patients [97, 98].

### Novel agents

Undiscovered and potentially confounding synergistic properties may of course be present with a multitude of other novel or repurposed agents. This is evident through preliminary reports of TTFields with Bevacizumab [62], as well as TTFields combined with Triflouropromazine, an approved antipsychotic drug. Triflouropromazine has been identified to inhibit mitotic slippage and yet did not decrease slippage when used in combination with TTFields [99, 100]. This is particularly interesting as the treatment appeared to decrease cell counts by up to 14% when used in combination, suggesting an improvement to efficacy independent of mitotic slippage. Cells treated in combination also experienced an increase in cell size of up to 35%, a well-documented phenomenon of TTFields [101], as well as a reduced clonogenic potential of the cells [99]. These results taken as a whole may encourage further investigation into TTFields in combination with novel and repurposed drugs.

More recently, TTFields were combined with Withaferin A [102], a steroidal lactone originating from the winter cherry plant, *Withania somnifera* [103]. Withaferin A had been previously shown to have efficacy against glioma cell lines *in vitro* as well as in murine orthotopic GBM models [104]. As has been a theme with other combinational therapeutic strategies with TTFields, greater efficacy is achieved when combining TTFields with Withaferin A compared to each treatment alone [102]. The mechanisms of Withaferin A have yet to be fully described, though reports have identified Withaferin A to affect expression of transcription factors, such as NF-κB [105]. NF-κB affects cytoskeletal assembly/disassembly [106], so this most likely one of the reasons why Withaferin A is also implicated in this process [107].

Interestingly, genome-wide expression analytical approaches are emerging for TTFields treated cell lines in order to further describe mechanisms of action, but also to attempt to characterise low-responsive vs high-responsive cell lines [108]. Karanam and colleagues provided data showing differential expression of multiple canonical pathways between responsive and low-responsive cell lines [108]. Producing similar data within brain tumour cell lines may be able to provide further direction towards more targeted combinations.

## CONCLUSIONS

This review has outlined and discussed the current literature on TTFields and its interactions with various therapeutic agents. However, given the limited efficacy of TTFields as a monotherapy [34], a need for a clear mechanism of action is apparent. There already exists ample descriptive preclinical studies at the cellular level for proposed mechanisms of action [12, 14], but there is a lack of mechanistic studies across more complex models with and without a combinational therapeutic approach. TTFields research is still in its relative infancy with ongoing research, endorsed by the success of the EF-14 trial. The main benefit of concurrent TTFields therapy is predominantly focussed on the lack of overlapping toxicities, however, reports of contact dermatitis is frequent and expected. Not to be overlooked is that TTFields does not appear to perpetuate any consequences synonymous with failed therapy i.e. promoting invasion and metastasis, although this has yet to be studied in-depth.

In conclusion, TTFields offers an exciting platform for a combinational therapeutic approach whether it is with novel or standard anti-tumour agents, with hopes that future treatment strategies may utilise these unique effects associated with alternating electric fields.
